# A Greedy Optimized Intelligent Framework for Early Detection of Alzheimer's Disease Using EEG Signal

**DOI:** 10.1155/2023/4808841

**Published:** 2023-02-22

**Authors:** R. Swarnalatha

**Affiliations:** Department of Electrical & Electronics Engineering, Birla Institute of Technology & Science, Pilani, Dubai Campus, Dubai, UAE

## Abstract

Recent researchers have been drawn to the analysis of electroencephalogram (EEG) signals in order to confirm the disease and severity range by viewing the EEG signal which has complicated the dataset. The conventional models such as machine learning, classifiers, and other mathematical models achieved the lowest classification score. The current study proposes to implement a novel deep feature with the best solution for EEG signal analysis and severity specification. A greedy sandpiper-based recurrent neural system (SbRNS) model for predicting Alzheimer's disease (AD) severity has been proposed. The filtered data are used as input for the feature analysis and the severity range is divided into three classes: low, medium, and high. The designed approach was then implemented in the matrix laboratory (MATLAB) system, and the effectiveness score was calculated using key metrics such as precision, recall, specificity, accuracy, and misclassification score. The validation results show that the proposed scheme achieved the best classification outcome.

## 1. Introduction

AD is a neurological malfunction identified by debilitation of analytic functions and amnesia [1]. This is mainly due to the unusual increase of protein around brain cells [2]. AD is often considered as a usual aging process and affects older people more than younger ones [3]. In humans, AD signs are increased confusion and loss of learning ability and memory [4]. Generally, AD is categorized into three stages based on its symptoms and effects [5]. In the beginning stage (mild AD), the sign of AD is most commonly amnesia [6], which does not change a person's daily life. The next phase is considered moderate AD [7], which is recognized by increased confusion and difficulty in learning [8]. In this phase, patient's dependence on the other people increases [9]. The third stage is severe AD, which is characterized by the entire debilitation of individual [10]. The early identification of AD is important in the case of mild AD and moderate AD [11, 12]. Detecting this disease is quite difficult because signs like amnesia are often considered as usual aging signs [13]. Moreover, the imaging and signal analysis system has been introduced in the digital field to analyze the AD severity range with the help of artificial intelligence (AI) [14, 15]. Hence, several neural models and classifiers were implemented to find the AD severity rate in the early stages without the requirement of high resource usage [16].

**Figure 1:**
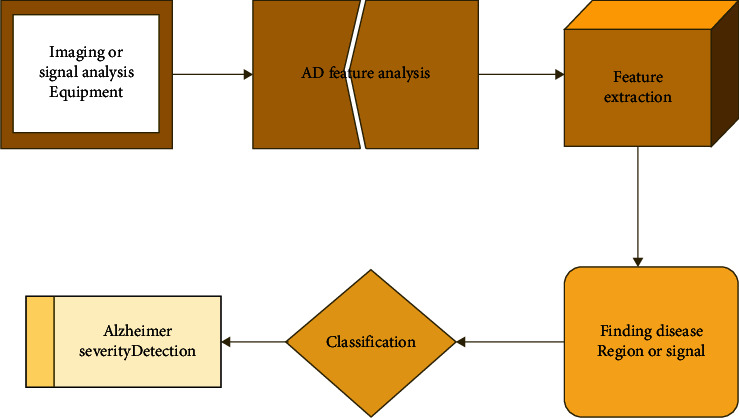
Basic Alzheimer detection using AI.

Generally, disease detection is done by large testing and eliminating the other disease cases [17]. Neurological testing, physiological and psychological examination, and blood test help to detect AD [18]. The basic Alzheimer's disease detection with AI is represented in Figure 1. Perform brain scans, such as computed tomography (CT), magnetic resonance imaging (MRI), or positron emission tomography (PET), to support an Alzheimer's disease diagnosis or rule out other possible causes for symptoms. Psychological testing such as mini-mental state testing and image techniques based on AI were developed to detect AD [19]. Still, they faced several demerits such as inaccurate disease classification, high error rate, and so on [20]. Thus, the researchers paid attention to AD detection techniques based on the analysis of EEG signals [21]. Here, the brain's reading is recorded using electrodes directly by 10–20 electrode systems [22]. AD diagnosis based on EEG signals provides early detection and accurate disease classification [23]. Recently, many researchers tried to detect AD in the early stage by identifying changes in the synchrony of EEG signals [24]. However, the diagnosis is not accurate in the case of single synchrony [25]. In addition, for the EEG signal-based AD severity analysis, different and several brain samples are taken from one person [26]. This has helped to find the brain neuron movements based on their actions and emotions. The EEG-based AD detection technique is used as a tool to diagnose AD early for a huge number of people [27].

To overcome the demerits faced by these techniques, a highly efficient and accurate AD detection was presented in this paper using EEG signals [28, 29]. In addition, a novel deep network has been executed by incorporating the optimal solution for detecting disease range. Also, in the designed model, the noise removal function is executed in the hidden phases that have afforded the finest noise feature tracking and neglecting. The present noises were eliminated properly in the filtering stage, so the algorithm complexity was minimized, and the classification score was maximized. Finally, a comparison assessment was conducted to check the improvement score in analyzing the AD severity range.

The current research is organized as follows.  Section 2 describes the existing works in detail. The problem related to analyzing the AD EEG signal is defined in Section 3. The proposed scheme and the solution of the problem are mentioned in Section 4. The proposed model's outcome is discussed in Section 5, and research arguments are concluded in Section 6.

## 2. Related Works

Some of the recent works related to AD detection are described below.

Safi and Safi [27] developed a technique by using Hjorth parameters to diagnose AD in its early stage. This model uses different filtering techniques such as empirical mode decomposition and discrete wavelet transform to filter the errors in the brain signals. Also, the *K*-nearest neighbor algorithm was applied to classify the diseases. In past decades, different neuropsychological testing based on magnetic resonance imaging (MRI) was developed to diagnose AD. Thus, Murugan et al. [28] developed a MRI-based convolutional neural network model to classify and detect AD accurately. This model is highly efficient in detection and classification of diseases. But, they did not provide accurate AD detection in case of a large population.

The interface between the features of EEG signal and AD is not clarified in existing detection models. Therefore, Li et al. [29] presented a novel technique to establish the relationship between AD detection and EEG signal which presents latent parameters in 3D phase and also provides high classification efficiency. However, the computational cost is high in this model. Deepa and Chokkalingam [30] presented an optimized VGG-16 framework to classify AD using arithmetic optimization which uses MRI-based images for disease classification. Here, the AD was classified as mild, moderate, and severe. Moreover, the computational complexity and cost are reduced in this technique. However, the experimental outcome of this technique for a large population does not provide efficient classification.

Recently, EEG signals have been widely used in medical fields to diagnose the disease in its early phase. Hence, Cejnek et al. [31] designed a model to detect mild AD based on EEG records. This technique uses an adaptive filter to preprocess the recorded EEG dataset. In predictive function, the linear NN technique with gradient adaption is used. This technique is validated by testing the data of AD patients. However, it does not attain high sensitivity and accuracy.

## 3. AD System Model and Problem Description

Usually, the disease forecasting process using signaling data is more complicated because of the feature variations in the minute level. Hence, the normal classification and signal analysis model has required more resources to find the feature variation and to identify the disease signal. In many cases, the algorithm complexity has been recorded because of signal analysis delay. These issues have motivated this work toward presenting an optimized deep neural system for EEG signal analysis and disease signal feature classification. The disease that was considered for validating the developed model is AD [32].

**Figure 2:**
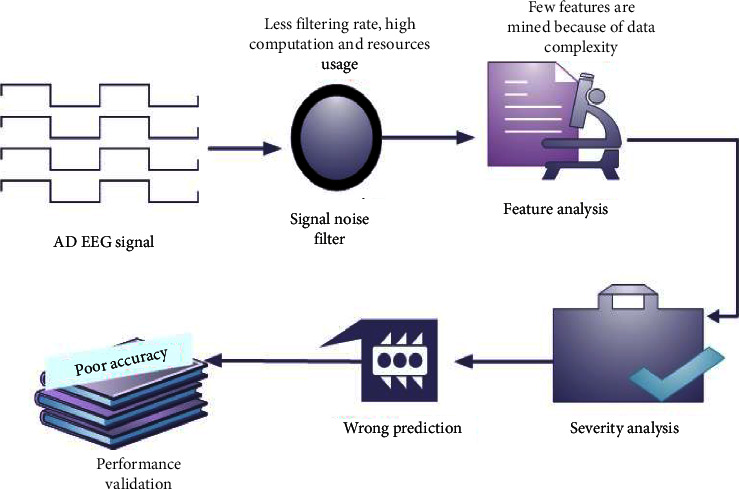
AD system model with problem.

Analyzing the brain signal is the foremost topic in the medical industry to detect and treat different kinds of diseases. Especially, analyzing the EEG signal and identifying the disease features and their severity range are highly complicated because of the noisy unbalanced data. The problems analyzing in EEG signal are described in Figure 2. A novel sandpiper-based recurrent neural system (SbRNS) has been introduced for predicting Alzheimer's disease in an earlier stage using EEG signal. Primarily, the EEG signals were collected and trained to the system and then the preprocessing function was activated to filter the present noise in the trained datasets. Consequently, the feature analysis was performed, and the disease features were extracted and then the severity range was measured. Finally, the key parameters were estimated and compared with the other schemes.

## 4. Proposed Methodology

**Figure 3:**
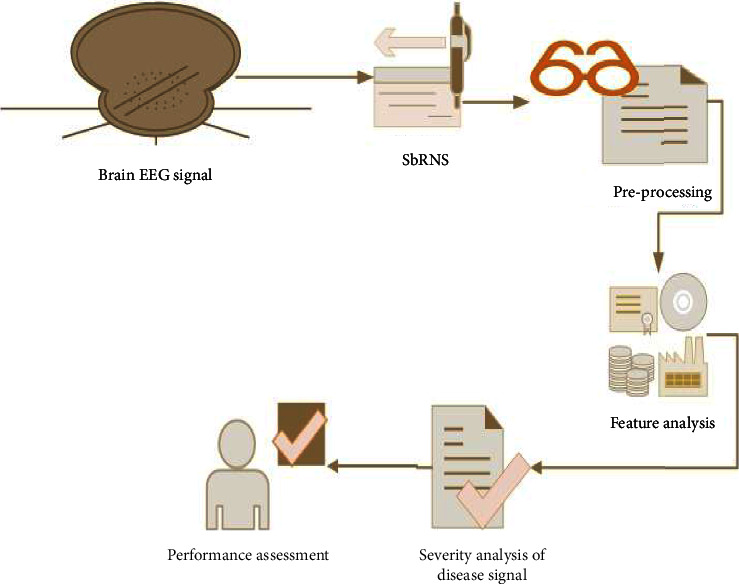
Proposed SbRNS architecture.

The proposed architecture is illustrated in Figure 3. In the feature analysis steps, the required features were traced and mined based on the specification of 0^th^ class and 1^st^ class.

Here, the signal pixels were analyzed and traced for the 0^th^ class features and then the extraction was done. Consequently, the disease specification and severity range estimation were performed. Finally, the comparison analysis helped to measure the performance enhancement by the developed scheme.

### 4.1. Proposed SbRNS Design

The planned model has five different phases that includes data importing, error filtering, classification module, optimal phase, and output phase. The data importing process is executed in the training phase of the initial layer. Noise elimination function is performed in the second phase hidden layer. Feature analysis and the severity level identification are processed in the third classification phase, and then the classification phase parameters are tuned by the optimal layer. In the fourth layer, the optimal phase sandpiper fitness is utilized. Finally, the severity forecasting outcome is recorded in the fifth layer, output phase.

**Figure 4:**
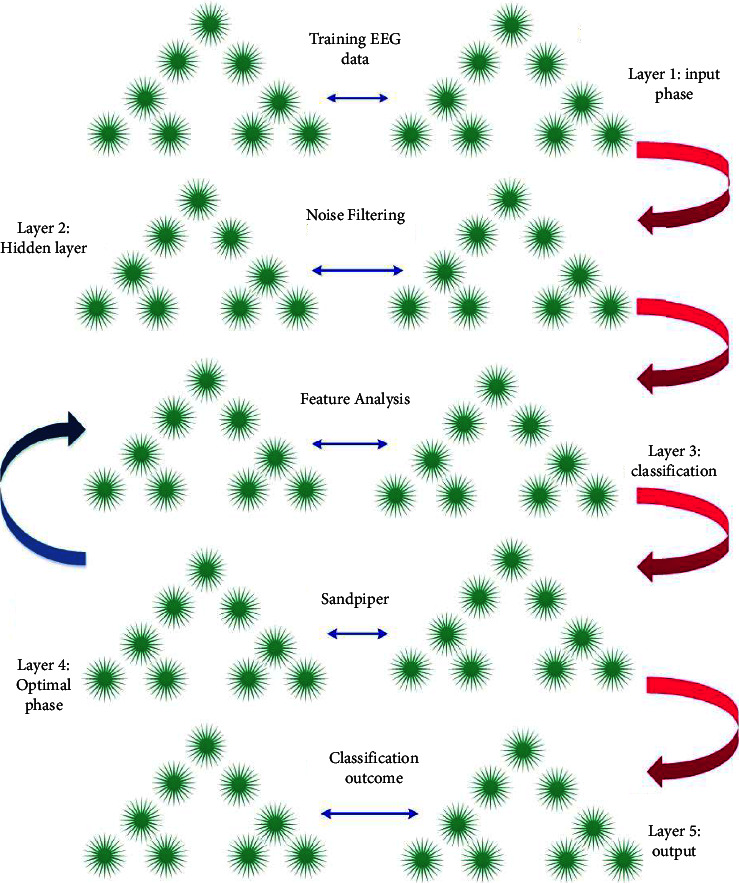
Layers of SbRNS.

The proposed scheme functions on the basis of the sandpiper approach [33] and recurrent neural scheme [34]. These layers are elaborated in Figure 4. Here, the reason for incorporating the sandpiper function in the recurrent neural classification phases is to earn the finest severity forecasting score.

### 4.2. Preprocessing Model

The function preprocessing is designed to eliminate the training flaws from the trained sets. This process tends to gain the finest accurate severity analysis score and less computational complexity. Moreover, the data importing process has been processed by (1). Here, the AD brain EEG signal dataset is determined as *b*_*e*_, dataset training function is determined as *F*(*b*_*e*_), and the *n* number of EEG signals is described as 1,2,3,4,…, *n*:

(1) $$\begin{array}{c} F\left(b_{e}\right)=b_{e}\left\{1,2,3,4,\cdots ,n\right\}\text{.} \end{array}$$

The data training function is performed in the input phase of the novel SbRNS. After training the data, the function preprocessing is activated in the hidden phase to eliminate the noise features.

(2) $$\begin{array}{c} J\left(b_{e}\right)=\left|b_{e}\left(q,a\right)-b_{e}\left(a\right)\right|\text{,} \end{array}$$

where *J* represents the preprocessing variable, *q* represents the normal features in the database, and the noise features are described as *a*. The noise features that are present in the trained database are removed by the preprocessing process that is represented as *b*_*e*_(*q*, *a*) − *b*_*e*_(*a*).

### 4.3. Feature Analysis

The output of the preprocessing layer is earned as the error-free data. Then, those data are considered as the input of the feature analysis process. Forecasting the disease or severity range in the entire data is difficult and has maximized the computational complexity. Considering this problem, the process feature analysis has been executed to track and extract the present features in the trained sets.

(3) $$\begin{array}{c} F^{*}\left(b_{e}\right)=Z-\left(\frac{y\left(b_{e}\right)}{\max}\right)\text{,} \end{array}$$

where the AD signal features are described as *Z* and *F*^*∗*^ determines the feature analysis variable. The process of feature extraction is indicated in (3). Here, *Z* represents the 0^th^ class. In this, the 0^th^ class features are traced and mined. Also, the unwanted features *y* are neglected because *y* falls under the 1^st^ class. In (3), max denotes the maximum possible iterations. This feature analysis process is executed by the fitness of sandpiper.

### 4.4. Classification Module

After the feature analysis function, the severity range of the AD has been predicted in the form of low, high, and medium. The analyzed features *Z* are categorized under the 0^th^ class and then AD range is specified as high severity; if *Z* falls under the 1^st^ class, then it is medium severity. If *Z* is not falling under classes 0 and 1, then it is specified as low severity.

(4) $$\begin{array}{c} D^{*}\left(b_{e}\right)=\left\{\begin{array}{cc} if\left(Z=0\right)\text{,} & high\text{,}\\ if\left(Z=1\right)\text{,} & medium\text{,}\\ else\text{,} & low\text{,} \end{array}\right. \end{array}$$

where *D*^*∗*^ represents the classification parameter. Hence, based on the “if” condition, the severity of the AD is specified as high and medium. The classification process is valued in (4).

**Figure 5:**
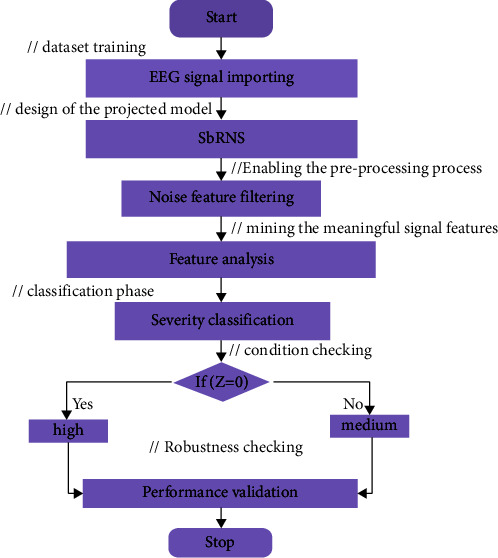
Flowchart of proposed SbRNS.

The defined formulation of the novel SbRNS is represented in Algorithm 1. By processing each step of SbRNS algorithm, the EEG signal was analyzed, and the severity score was forecasted. In addition, the flow of the steps in executing the present scheme is elaborated in Figure 5.

## 5. Results and Discussion

The discussed model is executed in the MATLAB programming platform and processed in Windows 10 platform. Initially, the EEG signal of AD is gathered from the standard site and taken as the input of the MATLAB system. The parameter specifications are indicated in Table 1.

In addition, the EEG signal processing framework functions in the ratio of 75 : 25, i.e., training 75% and testing 25%. The class that has been considered in the severity specification is low, medium, and high.

**Figure 6:**
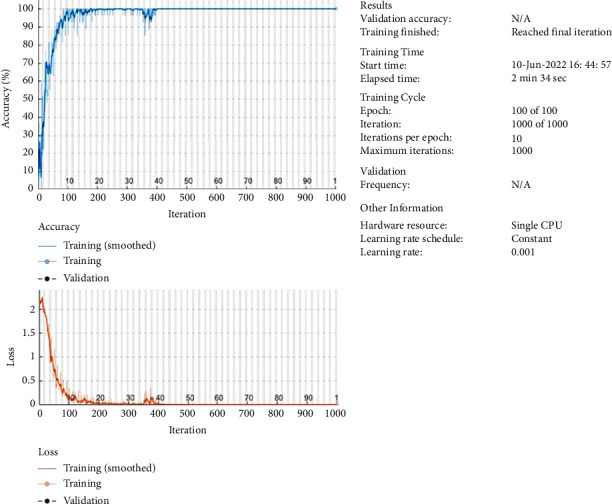
Validation of loss and accuracy.

The ability of the model in specifying the severity range is described through the validation, and the graphical representation is given in Figure 6. The validation graph has reached 100% accuracy range that described the exact prediction, and the loss is very low.

**Figure 7:**
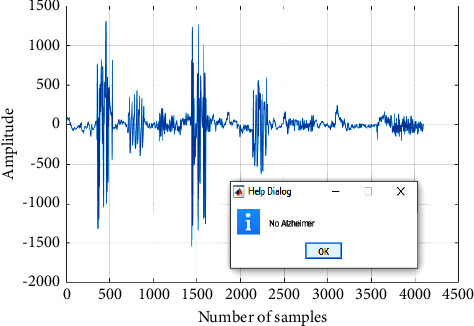
Normal EEG signal.

To measure the working range of the proposed design, a few different test samples are considered that are normal signal, low AD signal, medium AD signal, and high AD signal. Here, the normal signal is also considered for this experiment to check the working rate of the presented design. The results of the forecasted normal brain signal are shown in Figure 7.

**Figure 8:**
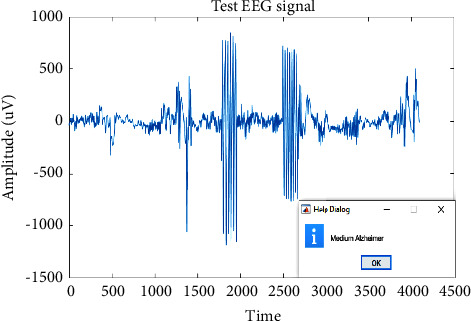
Classified medium severity AD.

**Figure 9:**
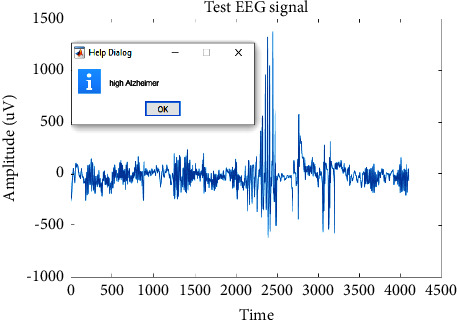
Classified high severity AD.

**Figure 10:**
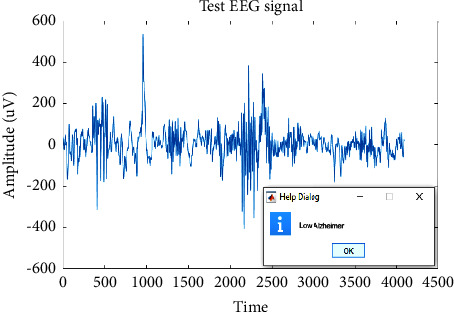
Classified low severity AD.

Different EEG signal samples were tested. The outcomes of medium AD, high AD, and low AD are indicated in Figures 8–10.

### 5.1. Performance Validation

Recall validation is an evaluation of the sensitivity range in classifying the severity categories. Also, it has provided the stability range of the executed model in classifying the disease range in the presence of false prediction and actual prediction. Thus, if a system has recorded a good recall measures, it is better in severity specification. The recall is was formulated as

(5) $$\begin{array}{c} recall=\frac{true\;positive\;}{false\;negative+true\;positive}\text{.} \end{array}$$

To measure the positive values in the severity prediction, the precision metrics were valued. Hence, this has afforded the actual forecasting outcome in severity range specification. The precision parameter is defined as

(6) $$\begin{array}{c} Precision=\frac{true\;positive\;}{false\;positive+true\;positive}\text{.} \end{array}$$

The exactness of severity specification is determined as accuracy. Hence, the gained accuracy has verified the robustness of the designed approach in specifying the Alzheimer severity range. The accuracy is defined as

(7) $$\begin{array}{c} accuracy=\frac{exact\;forecast}{total\;forecast}\text{.} \end{array}$$

The mean efficiency of the severity classification process has been found in the *F*-measure validation. The summation and product of the recall and precision have described the *F*-score, which is indicated as

(8) $$\begin{array}{c} F_{Value}=2\times \frac{recall\times precision}{recall+precision}\text{.} \end{array}$$

The metrics specification was estimated to assess the negative scores in predicting the AD severity range as follows:

(9) $$\begin{array}{c} specificity=\frac{true\;negative}{false\;positive+true\;negative}\text{.} \end{array}$$

**Figure 11:**
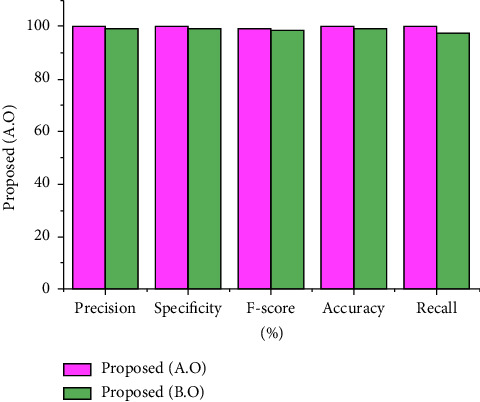
Performance assessment.

Here, the specificity score was determined by only taking into account negative classifications, i.e., a true negative and a false positive. Hence, the overall validation of the developed novel SbRNS was described in dual phases that are with and without incorporating the sandpiper fitness solution in the recurrent model, which is graphically defined in Figure 11. Before applying the sandpiper model in the recurrent system, the recorded specificity score was 98%; after adding the sandpiper function in the classification phase of the recurrent system, 99.5% of recall was recorded.

### 5.2. Comparison Assessment

The conventional schemes that have been taken used to validate the designed model are the extreme intelligent model (EIM) and *K*-nearest model (KNM) [35]. Besides, the presented approach is measured in dual phases that are before and after applying the optimization model.

The EIM has attained a precision score as of 99.69% and specificity of 99.6%; the approach KNM has earned a specificity rate as of 95.7% and precision of 96.7%. Moreover, before incorporating the sandpiper fitness, the presented scheme SbRNS has recorded the specificity score as of 99.3% and precision of 99.6%. After applying the fitness solution of sandpiper function model, 99.8% precision and 99.9% specificity were reported.

**Figure 12:**
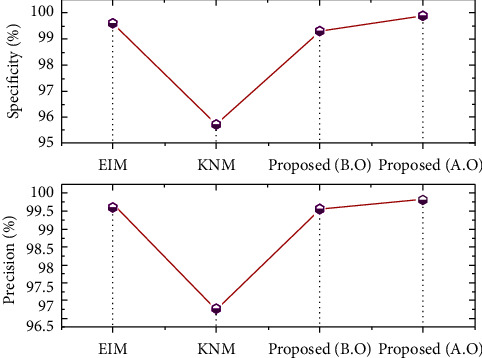
Assessment of specificity and precision.

The *F*-measure and accuracy rate gained by the scheme EIM are 98.9% and 98.7%, respectively; the approach KNM has gained an accuracy score of 98.1% and *F*-score of 98.3%. In addition, before performing the sandpiper function, the recorded *F*-score is 99% and accuracy is 99%. Furthermore, after executing the sandpiper model, the recorded *F*-score is 99.5%, and the exactness score is 99.8%. The statistics before and after the optimization are shown in Figure 12.

**Figure 13:**
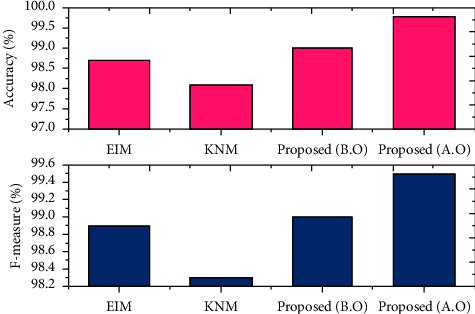
Assessment of accuracy and *F*-measure.

By comparing the recent scheme, the present approach has earned the best outcome in classifying the disease severity range. This has verified the effectiveness of the developed model in EEG signal analysis. In addition, after incorporating the sandpiper, the highest performance was recorded. It is clearly seen that the need of the optimal model in severity specification is highly recommended. The assessment of accuracy and *F*-measure is revealed in Figure 13. Also, the overall comparison assessment is described in Table 2.

### 5.3. Discussion

The validated parameters have described the proficient score of the designed model. The working performance of the proposed model is described by estimating all classification metrics that are precision, specificity, *F*-score, accuracy, recall, and error validation that are detailed in Table 3.

In this case, the metrics error was examined in order to examine the falling score of the designed approach. As a result, the earned misclassification score is only 0.1% percent, which is a very low and negligible state. This described the efficacy of the devised scheme.

## 6. Conclusion

A novel SbRNS has been implemented in the MATLAB framework for the classification of AD severity ranges using EEG signals, and the severity analysis procedure was carried out. The current SbRNS has a maximum severity forecast range of 99.8% when compared to other conventional approaches, and the classification rate was increased by 1%. The recorded sensitivity rate is 99.9%. In comparison to previous approaches, the specificity score has increased by 0.6% in comparison to earlier methods. The novel SbRNS was able to attain an extremely low error rate of 0.1%. Furthermore, when compared to the conventional recurrent neural model before applying the sandpiper function, the present SbRNS improved performance by 1% to 2%. As a result, the designed method is the a necessary scheme for EEG signal analysis and severity range prediction; however, it is not applicable to other application datasets. Creating a tuned hyper-parameter model will provide the best classification and feature analysis results in the future.

## Figures and Tables

**Algorithm 1:**
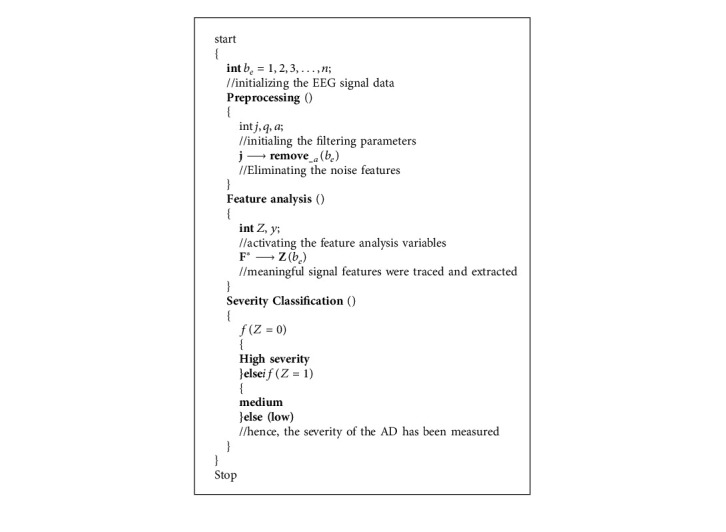
SbRNS.

**Table 1:** Details of execution parameters.

Parameter specifications	
Programming language	MATLAB
Version	R 2020 b
Operating system	Windows 10
Dataset type	AD signal
Signal type	EEG
Total EEG signals	11500
Objective	Severity range prediction

**Table 2:** Comparison assessment.

	Comparison statistics			
	Precision (%)	Specificity (%)	*F*-measure (%)	Accuracy (%)
EIM	99.69	99.6	98.9	98.7
KNM	96.7	95.7	98.3	98.1
SbRNS (B.O)	99.6	99.3	99	99
SbRNS (A.O)	99.8	99.9	99.5	99.8

**Table 3:** Performance assessment of SbRNS.

Overall validation	
Precision (%)	99.8
Specificity (%)	99.9
*F*-score (%)	99.5
Accuracy (%)	99.8
Recall (%)	99.95
Error (%)	0.1

## Data Availability

The data used to support the findings of this study are available online.

## References

[1] Metz C. N., Pavlov V. A. (2021). Treating disorders across the lifespan by modulating cholinergic signaling with galantamine. *Journal of Neurochemistry*.

[2] Martínez-Negro M., González-Rubio G., Aicart E., Landfester K., Guerrero-Martínez A., Junquera E. (2021). Insights into colloidal nanoparticle-protein corona interactions for nanomedicine applications. *Advances in Colloid and Interface Science*.

[3] Ferrari A., Stark D., Peccatori F. A. (2021). Adolescents and young adults (AYA) with cancer: a position paper from the AYA working group of the European society for medical oncology (ESMO) and the European society for paediatric oncology (SIOPE). *ESMO open*.

[4] Nguyen T. T., Nguyen T. T. D., Nguyen T. K. O., Vo T. K., Vo V. G. (2021). Advances in developing therapeutic strategies for Alzheimer’s disease. *Biomedicine & Pharmacotherapy*.

[5] Warren S. L., Moustafa A. A., Alashwal H. (2021). Harnessing forgetfulness: can episodic-memory tests predict early Alzheimer’s disease?. *Experimental Brain Research*.

[6] Thoe E. S., Fauzi A., Tang Y. Q., Chamyuang S., Chia A. Y. Y. (2021). A review on advances of treatment modalities for Alzheimer’s disease. *Life Sciences*.

[7] Tseng P. T., Jeng J. S., Zeng B. S. (2021). Efficacy of non-invasive brain stimulation interventions in reducing smoking frequency in patients with nicotine dependence: a systematic review and network meta-analysis of randomized controlled trials. *Addiction*.

[8] Knobler R., Arenberger P., Arun A. (2021). European dermatology forum: updated guidelines on the use of extracorporeal photopheresis 2020–Part 2. *Journal of the European Academy of Dermatology and Venereology*.

[9] Shanmugam J. V., Duraisamy B., Simon B. C., Bhaskaran P. (2022). Alzheimer’s disease classification using pre-trained deep networks. *Biomedical Signal Processing and Control*.

[10] Jayamohan H., Lambert C. J., Sant H. J. (2021). SARS-CoV-2 pandemic: a review of molecular diagnostic tools including sample collection and commercial response with associated advantages and limitations. *Analytical and Bioanalytical Chemistry*.

[11] Porsteinsson A. P., Isaacson R. S., Knox S., Sabbagh M. N., Rubino I. (2021). Diagnosis of early Alzheimer’s disease: clinical practice in 2021. *The journal of prevention of Alzheimer’s disease*.

[12] Dashwood M., Churchhouse G., Young M., Kuruvilla T. (2021). Artificial intelligence as an aid to diagnosing dementia: an overview. *Progress in Neurology and Psychiatry*.

[13] Alvi A. M., Siuly S., Wang H. (2021). Neurological abnormality detection from electroencephalography data: a review. *Artificial Intelligence Review*.

[14] Llerena Zambrano B., Renz A. F., Ruff T. (2021). Soft electronics based on stretchable and conductive nanocomposites for biomedical applications. *Advanced Healthcare Materials*.

[15] Alvi A. M., Siuly S., Wang H. (2021). Neurological abnormality detection from electroencephalography data: a review. *Artificial Intelligence Review*.

[16] Puri D., Nalbalwar S., Nandgaonkar A., Wagh A. (2022). EEG-based diagnosis of Alzheimer’s disease using Kolmogorov complexity. *Applied Information Processing Systems*.

[17] Khurana V., Gahalawat M., Kumar P. (2021). A survey on neuromarketing using eeg signals. *IEEE Transactions on Cognitive and Developmental Systems*.

[18] Rodrigues P. M., Bispo B. C., Garrett C., Alves D., Teixeira J. P., Freitas D. (2021). Lacsogram: a new EEG tool to diagnose Alzheimer’s disease. *IEEE Journal of Biomedical and Health Informatics*.

[19] Zhao X., Ang C. K. E., Acharya U. R., Cheong K. H. (2021). Application of Artificial Intelligence techniques for the detection of Alzheimer’s disease using structural MRI images. *Biocybernetics and Biomedical Engineering*.

[20] Qu Y., Wang P., Liu B. (2021). AI4AD: artificial intelligence analysis for Alzheimer’s disease classification based on a multisite DTI database. *Brain Disorders*.

[21] Gao Z., Dang W., Wang X. (2021). Complex networks and deep learning for EEG signal analysis. *Cognitive Neurodynamics*.

[22] Rahman M. M., Sarkar A. K., Hossain M. A. (2021). Recognition of human emotions using EEG signals: a review. *Computers in Biology and Medicine*.

[23] Wan Z., Yang R., Huang M., Zeng N., Liu X. (2021). A review on transfer learning in EEG signal analysis. *Neurocomputing*.

[24] Geng X., Li D., Chen H., Yu P., Yan H., Yue M. (2022). An improved feature extraction algorithms of EEG signals based on motor imagery brain-computer interface. *Alexandria Engineering Journal*.

[25] Guerrero M. C., Parada J. S., Espitia H. E. (2021). EEG signal analysis using classification techniques: logistic regression, artificial neural networks, support vector machines, and convolutional neural networks. *Heliyon*.

[26] Woodbright M., Verma B., Haidar A. (2021). Autonomous deep feature extraction based method for epileptic EEG brain seizure classification. *Neurocomputing*.

[27] Safi M. S., Safi S. M. M. (2021). Early detection of Alzheimer’s disease from EEG signals using Hjorth parameters. *Biomedical Signal Processing and Control*.

[28] Murugan S., Venkatesan C., Sumithra M. G. (2021). DEMNET: a deep learning model for early diagnosis of Alzheimer diseases and dementia from MR images. *IEEE Access*.

[29] Li K., Wang J., Li S. (2021). Feature extraction and identification of Alzheimer’s disease based on latent factor of multi-channel EEG. *IEEE Transactions on Neural Systems and Rehabilitation Engineering*.

[30] Deepa N., Chokkalingam S. P. (2022). Optimization of VGG16 utilizing the Arithmetic Optimization Algorithm for early detection of Alzheimer’s disease. *Biomedical Signal Processing and Control*.

[31] Cejnek M., Vysata O., Valis M., Bukovsky I. (2021). Novelty detection-based approach for Alzheimer’s disease and mild cognitive impairment diagnosis from EEG. *Medical, & Biological Engineering & Computing*.

[32] Chen Y., Strickland M. R., Soranno A., Holtzman D. M. (2021). Apolipoprotein E: structural insights and links to Alzheimer disease pathogenesis. *Neuron*.

[33] Kaur A., Jain S., Goel S. (2020). Sandpiper optimization algorithm: a novel approach for solving real-life engineering problems. *Applied Intelligence*.

[34] Lin J. C. W., Shao Y., Djenouri Y., Yun U. (2021). ASRNN: a recurrent neural network with an attention model for sequence labeling. *Knowledge-Based Systems*.

[35] Siuly S., Alçin Ö. F., Kabir E. (2020). A new framework for automatic detection of patients with mild cognitive impairment using resting-state EEG signals. *IEEE Transactions on Neural Systems and Rehabilitation Engineering*.

